# Semantic-Enhanced and Temporally Refined Bidirectional BEV Fusion for LiDAR–Camera 3D Object Detection

**DOI:** 10.3390/jimaging11090319

**Published:** 2025-09-18

**Authors:** Xiangjun Qu, Kai Qin, Yaping Li, Shuaizhang Zhang, Yuchen Li, Sizhe Shen, Yun Gao

**Affiliations:** 1 Aerospace Information Research Institute, Chinese Academy of Sciences, Beijing 100094, China; 2 School of Electronic, Electrical and Communication Engineering, University of Chinese Academy of Sciences, Beijing 101408, China

**Keywords:** autonomous driving, 3D object perception, sensor fusion, semantic information

## Abstract

In domains such as autonomous driving, 3D object detection is a key technology for environmental perception. By integrating multimodal information from sensors such as LiDAR and cameras, the detection accuracy can be significantly improved. However, the current multimodal fusion perception framework still suffers from two problems: first, due to the inherent physical limitations of LiDAR detection, the number of point clouds of distant objects is sparse, resulting in small target objects being easily overwhelmed by the background; second, the cross-modal information interaction is insufficient, and the complementarity and correlation between the LiDAR point cloud and the camera image are not fully exploited and utilized. Therefore, we propose a new multimodal detection strategy, Semantic-Enhanced and Temporally Refined Bidirectional BEV Fusion (SETR-Fusion). This method integrates three key components: the Discriminative Semantic Saliency Activation (DSSA) module, the Temporally Consistent Semantic Point Fusion (TCSP) module, and the Bilateral Cross-Attention Fusion (BCAF) module. The DSSA module fully utilizes image semantic features to capture more discriminative foreground and background cues; the TCSP module generates semantic LiDAR points and, after noise filtering, produces a more accurate semantic LiDAR point cloud; and the BCAF module’s cross-attention to camera and LiDAR BEV features in both directions enables strong interaction between the two types of modal information. SETR-Fusion achieves 71.2% mAP and 73.3% NDS values on the nuScenes test set, outperforming several state-of-the-art methods.

## 1. Introduction

With rapid technological advancements, precise 3D environment perception has become indispensable in areas such as autonomous driving, robotics, and smart cities. It serves as a core enabling technology for environmental understanding systems [1]. In the early stages of 3D perception research, most systems relied on single-modal data inputs, with cameras and LiDAR emerging as the two most widely adopted sensors and forming the technical foundation of that era. LiDAR-based methods [2,3,4,5,6,7] exploit the high-precision spatial representation capabilities of point cloud data, offering distinct advantages in object detection tasks. Nevertheless, they remain limited in fine-grained semantic analysis, particularly when classifying distant and small objects. Conversely, camera-based methods [8,9,10,11] demonstrate notable strengths in scene-level semantic interpretation; however, the inherent inability of 2D image data to accurately capture spatial and depth information constrains their localization accuracy.

To overcome the inherent limitations of individual sensors, some approaches [12,13,14,15] enhance the 3D perception accuracy by complementarily fusing LiDAR point clouds with 2D image data. These complementary modalities are usually characterized by two types of approaches: either by generating pseudo-point clouds from 2D image pixels via depth estimation and projecting them into the 3D space, or by projecting the point clouds or the derived proposals onto the image plane to extract corresponding 2D features. Both methods integrate multimodal information to enrich the 3D representations and improve semantic parsing. However, the first approach suffers from geometric distortions caused by depth estimation errors in occluded or textureless regions. The second method underutilizes dense image features by fusing only sparse point clouds or proposals, inevitably wasting rich semantic information.

**Figure 1:**
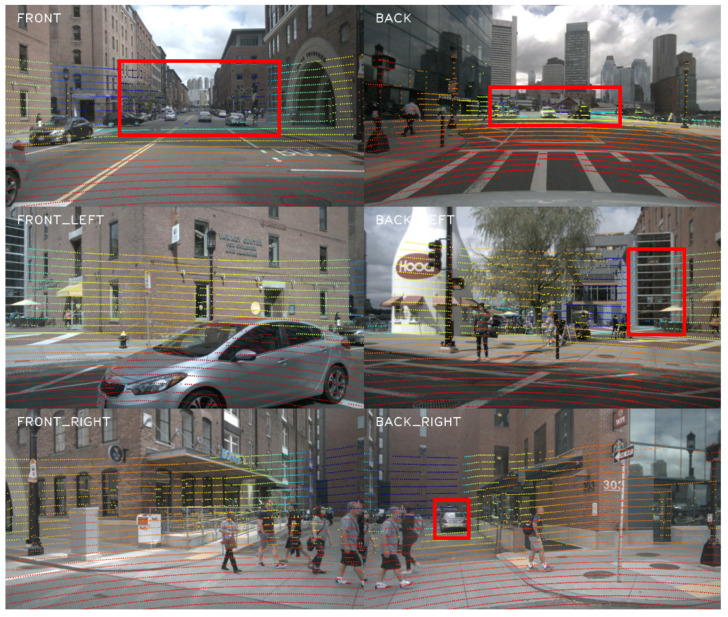
Distant point cloud thinning phenomenon. The red box in the figure highlights the absence of the LiDAR’s detection beam for distant objects.

Recently, state-of-the-art methods [16,17] have attempted to unify multimodal representations in bird’s eye view (BEV) space. The BEV perspective offers distinct advantages over 2D or 3D coordinate systems: it eliminates the scale variation and occlusion problems inherent in image views while facilitating downstream planning and control tasks. Although the BEV paradigm is gaining prominence in LiDAR–camera fusion, significant limitations persist. As illustrated in Figure 1, the small number of distant point cloud detections results in the loss of small target information on BEV features. Moreover, when fusing cross-modal features, this deficiency creates joint representations that neither fully preserve the LiDAR’s spatial precision nor adequately retain the camera’s semantic discrimination capability. Motivated by these challenges, we propose Semantic-Enhanced and Temporally Refined Bidirectional BEV Fusion (SETR-Fusion), a novel multimodal detection strategy.

**Figure 2:**
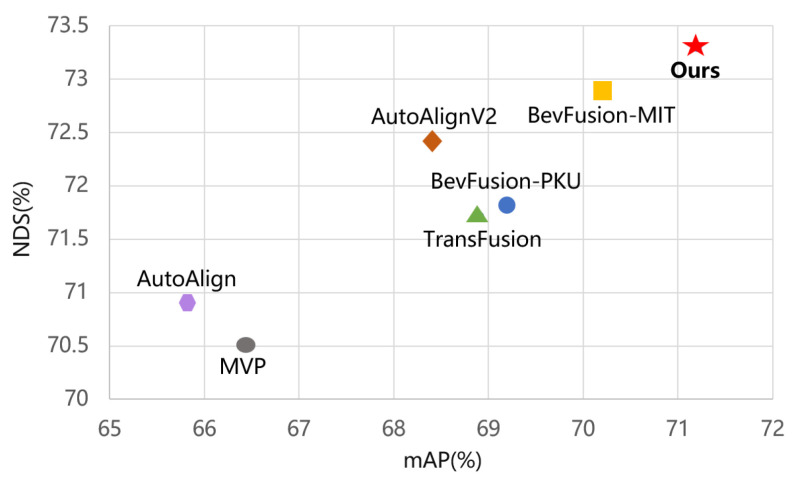
Performance comparison on the nuScenes test set. The detection performance of our model surpasses that of multiple existing models, demonstrating the effectiveness of the method.

To address the fine-grained perception problem of LiDAR, we introduce the Temporally Consistent Semantic Point Fusion (TCSP) module in the LiDAR stream. TCSP transfers the semantic features of image-based small targets to 3D point clouds, enhancing the perception of small objects. Meanwhile, in order to maximize the use of image semantic features, we introduce the Discriminative Semantic Saliency Activation (DSSA) module in the camera stream, which generates foreground and background response maps from semantic features and then fuses them with the image features to capture more discriminative foreground and background cues. This module effectively captures the salient features of small targets and suppresses the interference of complex backgrounds through a complementary learning approach. Finally, at the fusion stage, our Bilateral Cross-Attention Fusion (BCAF) fully interacts with and mutually enhances the information between different modalities. Our contributions are summarized as follows:

(1) We propose the TCSP module, which combines temporal consistency filtering with semantic annotation to significantly improve the distant target detection ability of sparse point clouds and alleviate the LiDAR fine-grained perception problem;
(2) We design the DSSA module, which enhances semantic discriminability through foreground–background response maps, amplifying small-target saliency while suppressing background interference;
(3) For multimodal fusion, we construct the BCAF module, which fully combines the image with the point cloud information to realize strong complementarity among multimodal features;
(4) Our model achieves 71.2% mAP and 73.3% NDS values on the nuScenes dataset, as shown in Figure 2, demonstrating the effectiveness of the proposed architecture.

## 2. Related Work

### 2.1. Single-Modal 3D Object Detection

Most of the early 3D object detection methods relied only on single-modal data from cameras or LiDAR. Camera-based 3D object detection can be categorized into two types: monocular and multiview detection. Various methods [18,19,20,21,22,23] focus on monocular detection, which lacks depth information and fails to model cross-view feature relationships, resulting in isolated multiview image representations. With the emergence of multiview image data [24,25], we can effectively solve the above problems. Some methods [9,10,26] use multiviews to generate dense 3D geometric representations for 3D target detection, but such methods lack a unified framework suitable for autonomous driving tasks. In contrast, BEV frameworks unify multiview imagery while preserving the geometric and semantic properties, providing explicit object localization and scale representation. Lift–Splat–Shoot (LSS) [12] independently lifts each camera image into feature-truncated cones, which are then Splat-fused to a unified BEV grid. BevDepth [27], BEVDet [28], and BevFormer [29] extract 2D features from multiview images and unify the multiview features in BEV space. BEVFormer [29], BevDet4D [30], PETRv2 [11], and StreamPETR [31] consider the temporal dimension by combining temporal modeling to achieve excellent detection performance. According to the different representations of point cloud processing, LiDAR-based 3D target detection is mainly categorized into point-based methods [32,33,34], which utilize a multilayer perceptron to directly process the point cloud data to obtain point features; voxel-based methods [2,3,7,35], which divide the point cloud into voxels and apply 3D sparse convolution to extract the voxel features; and pillar-based methods [4,36,37], which convert the raw point cloud into point pillars to extract features. Due to the inherent physical hardware limitations of a single sensor, unimodal-based 3D target detection is largely limited in terms of performance enhancement.

### 2.2. Multimodal 3D Object Detection

Multimodal fusion compensates for the shortcomings of single sensors by integrating complementary streams from cameras and LiDAR. This integration enables more robust and accurate target detection in complex driving scenarios. The current mainstream fusion strategies are mainly based on data mapping relationships. One class of approaches, as shown in [14,38,39,40], focuses on projecting image information into the LiDAR space: image pixels are projected into the point cloud coordinate system using the internal and external parameters of the camera to generate a “pseudo-point cloud”, where pseudo-points carrying image texture and semantic information are used to modify or enhance the feature representation of the original point cloud. In contrast, as described in [41,42], the LiDAR point cloud is projected onto the image plane to provide precise depth information to the corresponding image pixels, thus enhancing the understanding of the target spatial location by the image-based perceptual network.

However, the above unidirectional projection-based fusion methods share a common limitation: they all rely excessively on a single modality as the core vehicle for feature representation. This reliance leads to the inevitable loss or distortion of information from complementary modalities during the projection transformation process. For example, the projection of an image to a point cloud is susceptible to calibration errors, motion distortion, and occlusion, resulting in inaccurate pseudo-point locations or the loss of details, while the projection of a point cloud to an image often produces incomplete or noisy depth maps due to the sparseness of the point cloud. This loss of information limits the potential of the fusion effect. Recent studies [43,44,45] have clearly shown that realizing more symmetric and tighter co-fusion between the camera and LiDAR at the feature level can significantly improve the overall perceptual performance.

As a representative of this trend, BEVFusion [16,17] proposes an innovative fusion paradigm. Its core lies in using independent feature extraction branches to process image and point cloud data separately, fully preserving their respective modal properties. It utilizes geometric relations to transform and project the extracted image and point cloud features separately and uniformly into a shared BEV space. Under this spatially aligned unified coordinate system, the rich visual semantic information from the image and the precise geometric structure information from the point cloud can be fused to construct a powerful fused representation. Based on this fused representation, subsequent detectors can efficiently and accurately perform 3D target detection tasks. Although some progress has been made in multimodal fusion-based methods, there is still room for improvement in feature extraction and fusion methods.

Multimodal fusion methods, represented by BEVFusion, have achieved significant progress in 3D object detection, becoming a research hotspot. However, substantial room for improvement remains in feature extraction and fusion approaches. Recent advancements attempt to overcome these limitations from different angles: GAFusion [46] introduces LiDAR-guided global and local depth priors for adaptive fusion; SimpleBEV [47] offers a lightweight and efficient BEV fusion architecture; GraphBEV [43] achieves robust BEV feature alignment through local and global matching; CSDSFusion [48] offers a cross-supervision framework combining LiDAR-supervised depth estimation with multi-attention cross-fusion to mitigate semantic loss; IS-Fusion [49] introduces an instance–scene collaborative strategy, explicitly integrating instance-level multimodal features with scene-level BEV contextual information; DepthFusion [50] incorporates depth-aware guidance into hybrid fusion; ReliFusion [51] emphasizes reliability-based mechanisms to address sensor degradation issues; CL-fusionBEV [52] projects camera features into BEV space via implicit learning and fuses them with LiDAR through cross-attention and BEV self-attention; TiGDistill-BEV [53] employs LiDAR-to-camera distillation, transferring depth and BEV feature knowledge to boost monocular BEV detection; an attention-based fusion network [54] leverages channel-wise attention and a center-based BEV detector to improve geometric–semantic integration; and LDRFusion [55] adopts a LiDAR-dominant two-stage refinement strategy, generating proposals from LiDAR and enhancing them with pseudo-point clouds.

Despite this progress, these methods still face critical limitations: (1) constrained by sparse LiDAR point clouds, capturing distant small objects remains challenging even with geometric alignment; (2) most approaches fail to fully leverage dense semantic cues in images, limiting the recognition capabilities in cluttered backgrounds; (3) depth-guided or knowledge distillation strategies remain susceptible to estimation errors in occluded/textureless regions; (4) existing attention mechanisms or refinement methods predominantly focus on single-frame or unidirectional interactions, lacking deep bidirectional cross-modal collaboration and temporal semantic processing. Given the limitations of existing approaches, our work explores a more robust and discriminative 3D object detection method by integrating semantic augmentation, temporal optimization, and bidirectional BEV fusion techniques.

## 3. Methodology

**Figure 3:**
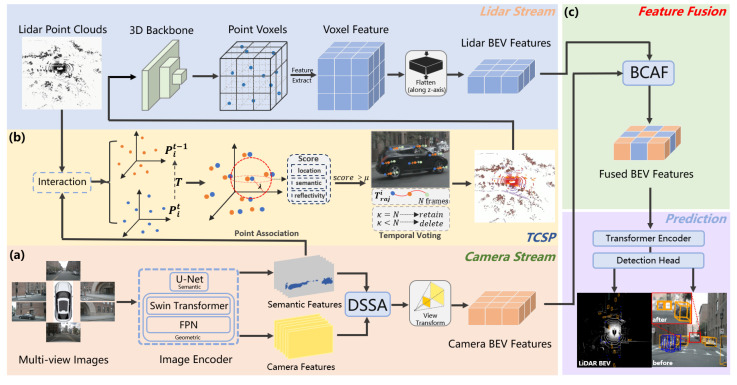
Overview of the SETR-Fusion framework. Subfigure (**a**) denotes the camera stream, subfigure (**b**) denotes the TCSP module, and subfigure (**c**) denotes the feature fusion module.

To address LiDAR’s difficulties in fine-grained perception and the shortcomings of the fusion strategy, we reconsider the significance of image semantic information and point cloud geometric information for detection. Our SETR-Fusion pipeline is shown in Figure 3. The proposed SETR-Fusion framework consists of two complementary processing branches, namely the camera stream and the point cloud stream. In the camera stream, multiview images are first processed by an image encoder to extract both semantic and geometric representations. These features are then refined through the DSSA module, which emphasizes salient foreground objects. Following a view transformation step, the enhanced representations are projected into the BEV space, yielding the camera BEV features. In the point cloud branch, the raw LiDAR point clouds are combined with semantic features from the images via a temporal consistency semantic point fusion module, which improves the detection of distant foreground targets. The fused point clouds are then processed by a 3D backbone to generate voxel-level features, which are subsequently projected along the Z-axis to obtain the LiDAR BEV features. Finally, the camera BEV features and LiDAR BEV features are fed into a bilateral cross-attention fusion module to achieve comprehensive multimodal BEV feature integration. The fused BEV features are subsequently passed through a detection encoder and a task-specific detection head to perform accurate 3D object detection.

### 3.1. Image Feature Extraction

**Figure 4:**
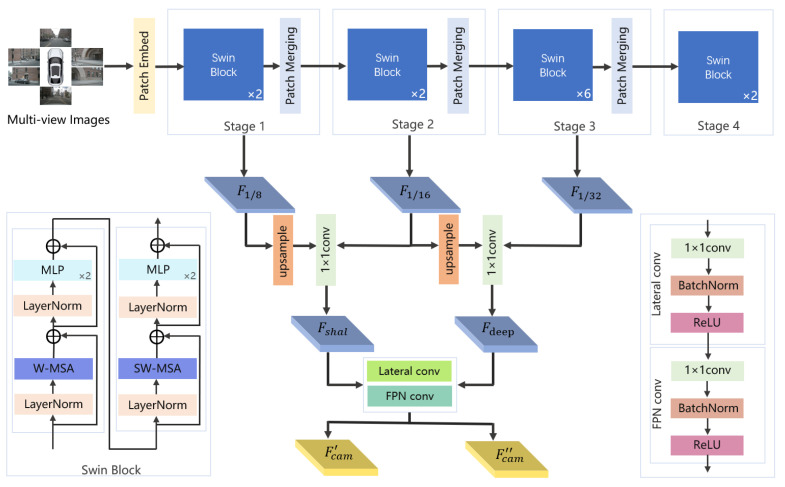
Image feature extraction structure.

In the image encoder of the SETR-Fusion camera stream, we design a dual-branch encoding architecture that jointly captures geometric and semantic information. Consider a set of input data $I\in R^{N_{C}\times H\times W\times 3}$, where $N_{C}$ is the number of viewpoints. In the semantic branch, a U-Net-based semantic segmentation network [56] is employed to extract instance-level segmentation features $F_{seg}\in R^{N_{C}\times H\times W\times K}$, where *K* denotes the number of object categories. In the geometric branch, we adopt a multiview image feature extraction framework based on a hierarchical Transformer architecture to process multiview image inputs in autonomous driving scenarios, as illustrated in Figure 4. Specifically, the feature extraction pipeline first flattens the multiview images into six independent samples, which are then fed into a Swin Transformer backbone [57] with shared weights. The core computational unit of this backbone is the Swin block, which incorporates a hierarchical window-based self-attention mechanism to achieve efficient long-range dependency modeling. Each Swin block contains a shifted window multihead self-attention module with layer normalization, which alternates between local attention computation through fixed-window partitioning and shifted-window strategies, thereby enabling cross-window feature interaction. To mitigate overfitting, DropPath regularization is applied. Following the attention module, a feedforward network is employed, consisting of a 4× channel-expanded MLP with GELU activation, further enhanced by residual connections to improve the information flow. This cascaded “attention + feedforward” design preserves global context awareness while significantly reducing the computational complexity.

The backbone network processes features through four hierarchical stages. The initial patch embedding module employs a 4×4 convolution to downsample the input, producing the initial feature maps. Subsequently, four sequential processing stages progressively reduce the spatial resolution while increasing the representational capacity, generating a set of multiscale features $F_{1/8}$, $F_{1/16}$, and $F_{1/32}$. These multiscale outputs are fused through a feature recomposition module. The shallow-level features $F_{shal}$ are obtained by concatenating the outputs of stages 1 and 2 after upsampling $F_{1/8}$ to match the resolution of $F_{1/16}$, followed by a 1×1 convolution for channel reduction. Similarly, the deep-level features $F_{deep}$ are generated by fusing the outputs of stages 3 and 4 and applying a 1×1 convolution for dimension reduction. The reorganized features are fed into the neck module, a lightweight variant of the Feature Pyramid Network (FPN) [58]. This module first compresses both shallow and deep features to a uniform channel dimension *C* via 1×1 convolutions, followed by 3×3 convolutions to enhance spatial context modeling. Finally, it outputs multiscale feature pairs $F_{cam}^{\prime }\in R^{N_{C}\times H\times W\times C}$ and $F_{cam}^{\prime }\in R^{N_{C}\times H\times W\times C}$ with consistent channel dimensions, where *C* is the number of feature channels. In this design, high-resolution features preserve fine-grained spatial details, whereas low-resolution features encode high-level semantic cues, enabling balanced and optimized representations for multiview 3D perception.

Overall, the hierarchical attention mechanism within the Swin blocks facilitates cross-window information interaction, while the feature recomposition strategy balances computational efficiency with multiscale expressiveness, forming an end-to-end feature extraction pipeline.

### 3.2. Discriminative Semantic Saliency Activation

**Figure 5:**
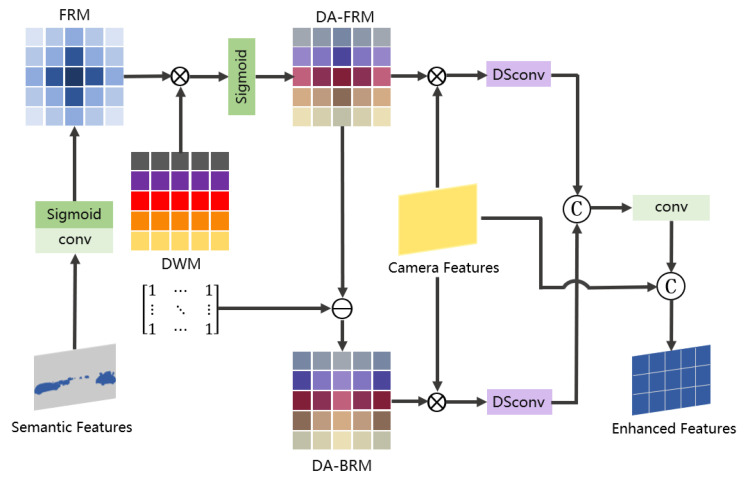
Performance comparison on the nuScenes test set.

Images provide dense semantics that are critical for contextual understanding and precise foreground–background differentiation. To fully leverage this capability, we enhance the camera branch with the DSSA module, which explicitly fuses semantic and geometric image features. This design maximizes the use of semantic cues and significantly improves the perception performance, especially for distant small targets near the sensor’s range limits. The camera branch pipeline is shown in Figure 3a, and the details of the DSSA module are demonstrated in Figure 5.

**Figure 6:**
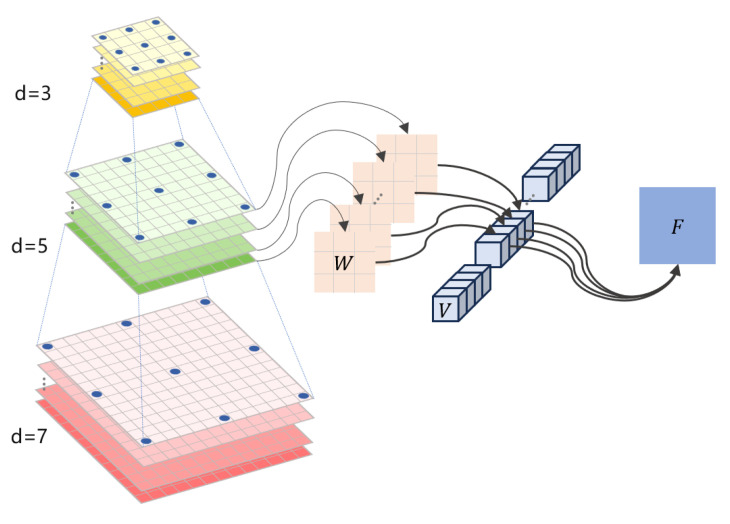
Depth separable expansion convolution.

$F_{cam}\in R^{N_{C}\times H\times W\times C}$ and $F_{seg}\in R^{N_{C}\times H\times W\times K}$ are obtained by the image encoder, where *C* is the number of feature channels and *K* is the number of categories. Inputting $F_{cam}$ and $F_{seg}$ into the DSSA module, $F_{seg}$ generates the foreground response map (FRM) of the emphasized instances after 1×1 convolution and Sigmoid; then, we refer to [59] to generate the image into a depth weight map (DWM). We then multiply the FRM with the DWM element-by-element pointwise to obtain the depth-augmented foreground response map (DA-FRM); finally, the depth-augmented background response map (DA-BRM) can be obtained by subtracting the DA-FRM with the all-one matrix. Next, the DA-FRM and DA-BRM are weighted with $F_{cam}$ to obtain $C_{F}\in R^{N_{C}\times H\times W\times C/4}$ and $C_{B}\in R^{N_{C}\times H\times W\times C/4}$, respectively, integrating fine-grained details to enhance the characterization and discriminative properties of small targets in the feature mapping. To enhance the camera’s ability to perceive distant and small instances, as shown in Figure 6, we incorporate the depth-separable dilation convolution $D_{i}^{DS}$ [60]. This module sequentially comprises three steps. First, dilated convolutions are performed independently on each channel through per-channel dilated convolutions. Different dilation rates significantly expand the receptive field while maintaining manageable computational complexity, enabling the capture of contextual information across varying scales. Second, deep convolutional kernels are introduced to perform further spatial feature extraction and enhancement on the per-channel features; finally, 1 × 1 pointwise convolutions are employed to achieve cross-channel linear combinations and information fusion, thereby enhancing the overall expressive power of the feature representations. This architecture maintains multiscale receptive field coverage while effectively reducing the computational complexity of traditional convolutions. It improves the detection performance for small objects at near, medium, and far distances. It is detailed in the following formula:

(1) $$D_{i}^{DS}\left(C\right)=\left\{\begin{array}{c} ReLU(Conv(1\times 1))(i=1)\\ ReLU\left(V_{i}*\left({\hat{W}}_{i}*C\right)\right)\left(i=2,3,4\right) \end{array}\right.$$

where $D_{i}^{DS}\left(C\right)$ consists of a 1×1 convolution kernel, 256 channels, and a layer of ReLU when i=1. When i=2,3,4, $D_{i}^{DS}\left(C\right)$ is a depth-separated convolution with expansion rates of 3, 5, and 7, respectively, and the number of channels is 256, where $V_{i}\in R^{C_{out}\times C_{in}\times 1\times 1}$ is a 1×1 convolution kernel, ${\hat{W}}_{i}\in R^{C_{in}\times 1\times K_{S}\times K_{S}}$ denotes a depth-separated convolution kernel independent of each group of channels, $K_{S}$ represents the size of the convolution kernel, and *C* denotes the input data. The small target scale is widely distributed, and it is difficult to take into account the single receptive field structure. The design covers 7×7 to 15×15 receptive fields with different expansion rates, realizing the capture of different contextual information features at near, middle, and far distances. With inputs $C_{F}$ and $C_{B}$, the outputs with different expansion rates are concatenated and output using $D_{i}^{DS}\left(C\right)$ to obtain $G_{0}$. Finally, $G_{0}$ is then connected in series with $F_{cam}$ to obtain the enhanced image features $F_{enh}\in R^{N_{C}\times H\times W\times C}$ through the 3×3 kernel and ReLU layer.

(2) $$G_{0}=L\left(\left[{⊔}_{i=1}^{4}D_{i}^{DS}\left(C_{F}\right),{⊔}_{i=1}^{4}\;D_{i}^{DS}\left(C_{B}\right)\right]\right)$$

(3) $$F_{enh}=\left[G_{0},F_{cam}\right]$$

where ⊔ denotes the join operation, *L* denotes the output layer, and [,] denotes the tandem operation.

### 3.3. Camera BEV Feature Construction

We adopt an implicitly supervised approach to construct camera BEV features, with the goal of transforming the image data into spatially consistent feature representations by predicting the depth distribution of each pixel. This strategy projects rich image features into appropriate depth intervals in three-dimensional space, thereby generating BEV representations that are tightly coupled with the object structure and depth cues. Such representations provide high-quality feature inputs for downstream tasks such as localization, navigation, and 3D detection.

**Figure 7:**
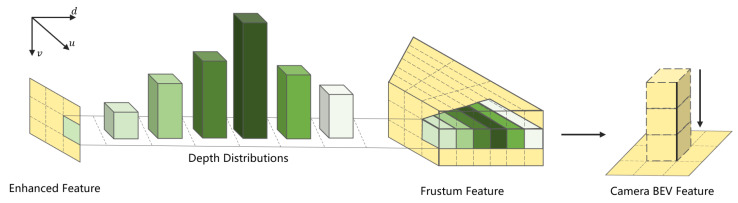
Camera BEV feature generation process.

Specifically, as shown in Figure 7, the extracted image features $F_{enh}$ are fed into the camera BEV encoder, which integrates the Lift–Splat–Shoot (LSS) [12] method to predict per-pixel depth distributions. This enables the more accurate estimation of each pixel’s position and depth in 3D space, forming a robust foundation for subsequent feature transformation. Each image feature point is then sampled along the corresponding camera ray into multiple discrete depth hypotheses, where the predicted depth distribution defines a probability density function for each pixel. Based on these probabilities, each feature point is rescaled according to its depth value, ensuring a precise spatial arrangement and ultimately forming a feature point cloud in 3D space.

Once the feature point cloud is obtained, it is compressed along the depth axis to aggregate the information into a compact BEV representation $B_{I}\in R^{H\times W\times C_{I}}$, where $C_{I}$ denotes the BEV feature channel dimension. This compression step removes redundant depth variations while preserving the most informative geometric and spatial structural cues. The resulting BEV feature map effectively encodes both geometric and semantic information from the original images in a 3D spatial context, providing an efficient and discriminative representation for downstream visual perception tasks.

### 3.4. Temporally Consistent Semantic Point Fusion

In order to compensate for the shortcomings of LiDAR for distant target detection, we insert a temporal consistency semantic point fusion module before the point cloud enters the LiDAR branch, aiming to enhance the detection ability of the point cloud for the foreground, and its pipeline is shown in Figure 3b.

**Point Association.** Semantic feature points are fused with the point cloud $P_{i}\in R^{4}$ with reference to [14,61] to generate semantic LiDAR points $P_{i}^{S}\in R^{4\times c\times s}$, where the number 4 contains the point cloud coordinates (X,Y,Z) and the intensity *r*, *c* is the category, and *s* is the confidence. The real objects have continuous motion trajectories in the time dimension, while the noise points appear randomly. In order to make the fusion results more accurate, the semantic LiDAR points are filtered for temporal consistency. Firstly, we obtain the positional transformation matrix *T* between neighboring frames and transform the current frame point cloud $P_{i}^{t}$ into the previous frame point cloud $P_{i}^{t-1}$ coordinate system. Second, KD-Tree is utilized to establish correspondence between the current frame points and the historical frame points, and we search for points that match the radius λ as candidate points. Finally, according to the distance, semantic confidence, and radar reflectivity, the candidate points are assigned a location similarity score $Sim_{pos}$, semantic similarity score $Sim_{sem}$, and reflectivity similarity score $Sim_{ref}$, respectively, which are computationally defined as follows:

(4) $$Sim_{pos}=e^{-\frac{\left|\Delta p\right|}{\sigma_{p}}}$$

(5) $$Sim_{sem}=I\left(Same_{class}\right)$$

(6) $$Sim_{ref}=1-\left|\Delta r\right|$$

where |Δp| is the Euclidean distance between the current point and the candidate point, and $\sigma_{p}$ is the distance scale parameter. *I* is a binary indicator function that satisfies the same semantic category, outputting 1; otherwise, it is 0. |Δr| is the absolute difference between the reflectance value of the current point and that of the candidate point. From the above, the scoring model is built:

(7) $$Score=w_{p}\cdot Sim_{pos}+w_{s}\cdot Sim_{sem}+w_{r}\cdot Sim_{ref}$$

where $w_{p}$ is the spatial distance weight, $w_{s}$ is the category invariance weight, and $w_{r}$ is the material consistency weight.

**Temporal Voting.** We set the score threshold μ and the list of temporal point trajectories $T_{raj}^{i}$. From the comprehensive scores of candidate points, we determine whether there is a best candidate point among the candidate points. If so, we deposit it into the trajectory $T_{raj}^{i}$; if not, we consider it as a new point. After consecutive *N* frames, we determine whether the length of the trajectory $T_{raj}^{i}$ is greater than κ. If κ is equal to *N*, the current frame point is retained; if it is less than κ, the current frame point is regarded as noise and rejected. Finally, the enhanced point cloud $P_{enh}$ filtered by temporal consistency is obtained. The TCSP module’s algorithmic flow is shown in Algorithm 1.

Next, we input $P_{enh}$ into the LiDAR branch, which we first divide into regular voxels $V_{p}\in R^{X_{V}\times Y_{V}\times Z_{V}}$ and extract voxel features using a voxel encoder [3] with 3D sparse convolution. Then, we project the voxel features to the BEV along the z-axis and use multiple 2D convolutional layers to obtain the LiDAR BEV feature map $B_{p}\in R^{H\times W\times C_{L}}$, where $C_{L}$ is the number of LiDAR BEV feature channels.

**Algorithm 1** Temporally Consistent Semantic Point Fusion**Require:** Current frame semantic LiDAR points $P_{i}^{S}\in R^{4\times c\times s}$, previous frame active   trajectories active_traj, transformation matrix *T* (current to previous frame), parameters:   λ (radius), $\sigma_{p}$ (distance scale), weights: $w_{p}$, $w_{s}$, $w_{r}$, thresholds:   μ (score), κ (trajectory length), persistent state: next_id (trajectory counter) **Ensure:** Enhanced point cloud $P_{\mathrm{enh}}$, updated active trajectories updated_active_traj, updated   next_id
1:$P_{\mathrm{enh}}\;\leftarrow \;\emptyset$, updated_active_traj←∅, extended_trajs←∅, new_trajs←∅2:**for** each point $p\in P_{i}^{S}$ **do**3:   Compute $\hat{p}\leftarrow T\cdot p$4:**end for**5:T ← Build_KDTree({tr.last_point∣∀tr∈active_traj})6:**for** each point $p\in P_{i}^{S}$ (with transformed $\hat{p}$) **do**7:   $\mathrm{candidates}\leftarrow \mathrm{KDTree}\_\mathrm{RadiusSearch}(T,\hat{p},\lambda )$8:   **if** candidates exists **then**9:       best_score←−∞, best_tr←None10:      **for** each trajectory tr∈candidates **do**11:         $\Delta p\leftarrow ∥{\hat{p}}_{\mathrm{xyz}}-\mathrm{tr}.\mathrm{last}\_{\mathrm{point}}_{\mathrm{xyz}}{∥}_{2}$12:         ${\mathrm{Sim}}_{\mathrm{pos}}\leftarrow e^{-\Delta p/\sigma_{p}}$13:         ${\mathrm{Sim}}_{\mathrm{sem}}\leftarrow \left\{\begin{array}{ccc} 1 & \mathrm{if}\;p_{\mathrm{class}}=\mathrm{tr}.\mathrm{last}\_{\mathrm{point}}_{\mathrm{class}}0 & \mathrm{otherwise} \end{array}\right.$14:         $\Delta r\leftarrow |p_{r}-\mathrm{tr}.\mathrm{last}\_{\mathrm{point}}_{r}|$15:         ${\mathrm{Sim}}_{\mathrm{ref}}\leftarrow 1-\Delta r$16:         $\mathrm{score}\leftarrow w_{p}\cdot {\mathrm{Sim}}_{\mathrm{pos}}+w_{s}\cdot {\mathrm{Sim}}_{\mathrm{sem}}+w_{r}\cdot {\mathrm{Sim}}_{\mathrm{ref}}$17:         **if** score>best_score **then**18:           best_score←score, best_tr←tr19:         **end if**20:      **end for**21:      **if** best_score≥μ **then**22:         Update best_tr.last_point←p (current frame coordinates)23:         best_tr.length←best_tr.length+124:         Add best_tr to extended_trajs25:         **if** best_tr.length≥κ **then**26:           $P_{\mathrm{enh}}\leftarrow P_{\mathrm{enh}}\cup \left\{p\right\}$27:         **end if**28:         **continue**29:      **end if**30:   **end if**31:   Create new trajectory:32:    new_tr←{id:next_id,last_point:p,length:1}33:   next_id←next_id+134:   Add new_tr to new_trajs35:   **if** 1≥κ **then** $P_{\mathrm{enh}}\leftarrow P_{\mathrm{enh}}\cup \left\{p\right\}$36:   **end if**37:**end for**


### 3.5. Bilateral Cross-Attention Fusion

Recent studies [16,17] have constructed shared BEV representations through simple feature concatenation. However, these approaches suffer from fundamental limitations: on the one hand, no cross-modal interaction mechanism has been established, leading to the isolation of geometric and semantic information; on the other hand, the lack of global spatial correlation modeling degrades feature fusion to a local operation. This coarse-grained fusion fails to satisfy complementary modality requirements in dynamic scenes. For this reason, we propose the BCAF mechanism in the cross-modal perception task, which realizes a strong synergy between point clouds and image modalities through symmetric feature interaction.

**Figure 8:**
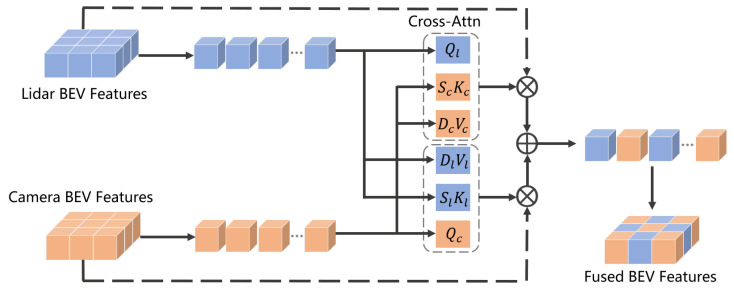
The architecture of BCAF.

As shown in Figure 8, the framework first adds position encoding $P_{ec}$ and $P_{el}$ to the image BEV feature sequence $F_{bc}^{seq}$ and the point cloud BEV feature sequence $F_{bl}^{seq}$ to preserve spatial information, respectively. The cross-attention formula is as follows:

(8) $$CrossAttn\left(Q,K,V\right)=Softmax\left(\frac{QK^{T}}{\sqrt{d_{k}}}\right)V$$

where Q, K, and *V* denote the query, key, and value, respectively. We create low-rank projection matrices $S_{c}$, $S_{l}$ and $D_{c}$, $D_{l}$, respectively, for the two attention directions, where rank r=64. Each C×C high-density projection of *K* and *V* is replaced with low-rank projections C×r and r×C, reducing the parameters and floating-point operations of the linear terms from $C^{2}$ to 2Cr and lowering the computational complexity. The core design of the BCAF module consists of two parallel cross-attentional branches, formulated as follows:

(9) $$Q_{c}=F_{bc}^{seq}+P_{ec},K_{l}=F_{bl}^{seq}+P_{el},V_{l}=F_{bl}^{seq}+P_{el}$$

(10) $$F_{c2l}=CrossAttn\left(Q_{c},S_{l}K_{l},D_{l}V_{l}\right)$$

(11) $$Q_{l}=F_{bl}^{seq}+P_{el},K_{c}=F_{bc}^{seq}+P_{ec},V_{c}=F_{bc}^{seq}+P_{ec}$$

(12) $$F_{l2c}=CrossAttn\left(Q_{l},S_{c}K_{c},D_{c}V_{c}\right)$$

where $Q_{c}$ is the query vector of the image modality, and $K_{l}$ and $V_{l}$ denote the key and value vectors of the point cloud modality, respectively. $F_{c2l}$ denotes the image BEV features enhanced using the point cloud information. $Q_{l}$ denotes the query vector of the point cloud modality, and $K_{c}$ and $V_{c}$ denote the key and value vectors of the image modality, respectively. $F_{l2c}$ denotes the point cloud BEV features enhanced using the image information. Finally, the bilaterally enhanced features are fused into a unified representation:

(13) $$F_{fused}=Concat\left(F_{c2l},F_{l2c}\right)$$

### 3.6. Loss Function

**DSSA Module Loss.** The DSSA module partitions BEV-space features into foreground and background channels to enhance the discriminative capabilities of multimodal fusion features. Let the foreground saliency map predicted by the DSSA module be denoted as $S_{fg}$ and the background saliency map as $S_{bg}$. The binary mask $M_{fg}$ is obtained by projecting the ground truth segmentation labels of instances within 3D bounding boxes onto the BEV space. Specifically, for each annotated object, we first rasterize its 3D bounding box onto the BEV grid, marking grid cells within the object region as foreground (1) and those outside as background (0). The resulting binary mask $M_{fg}$ provides explicit supervision signals for the foreground saliency map $S_{fg}$ and background saliency map $S_{bg}$. To address class imbalance and place a greater emphasis on hard-to-classify samples, we employ the binary focal loss [62] to supervise the foreground and background predictions separately:

(14) $$L_{fg}=-\frac{1}{Z}\sum_{u}M_{fg}\left(u\right){\left[1-S_{fg}\left(u\right)\right]}^{\gamma }logS_{fg}\left(u\right)$$

(15) $$L_{bg}=-\frac{1}{Z}\sum_{u}M_{bg}\left(u\right){\left[1-S_{bg}\left(u\right)\right]}^{\gamma }logS_{bg}\left(u\right)$$

where α=0.25 and γ=2.0 are pixel normalization factors. Finally, the loss of the DSSA module is

(16) $$L_{DSSA}=\lambda_{fg}L_{fg}+\lambda_{bg}L_{bg}$$

where $\lambda_{fg}$ and $\lambda_{bg}$ are loss weight hyperparameters.

**TCSP Module Loss.** The TCSP module achieves time-consistent semantic point fusion by calculating the cross-frame association score between the current frame and historical frame points. Let the matching score between the current frame point $P_{i}^{t}$ and the historical frame candidate point $P_{i}^{t-1}$ be $S_{ij}$, which is a weighted sum of the spatial position similarity, semantic feature similarity, and reflectance similarity. To ensure matching quality, we introduce a ranking consistency loss constraint on the interval between positive and negative sample scores:

(17) $$L_{rank}=\frac{1}{|P^{+}|}\sum_{\left(i,j^{+}\right)}\frac{1}{|N^{+}|}\sum_{j^{-}\in N_{i}^{-}}max\left(0,m_{s}-S_{ij^{+}}+S_{ij^{-}}\right)$$

where $P^{+}$ is the set of point pairs in which the association between a current-frame point and a historical-frame point satisfies both the spatial distance threshold and the semantic category consistency criterion. The notation $|P^{+}|$ is the total number of positive matching pairs. *i* is the current index, $j^{+}$ is the historical-frame positive sample point matching the current-frame point *i*, and $N_{i}^{-}$ is the set of negative samples for the current-frame point *i*. $|N_{i}^{-}|$ is the number of negative samples for the current-frame point *i*, $j^{-}$ is the index of the negative matching point for the current-frame point, $S_{ij}$ is the cross-frame matching score between the current-frame point *i* and the historical-frame point *j*, and $m_{s}$ is the hyperparameter for the matching score interval, used to widen the gap between positive and negative matching scores.

At the same time, to avoid jumps in the trajectory after cross-frame fusion, a trajectory smoothing loss is introduced to constrain the continuity of the matching points in adjacent frames, and a reflectance consistency term is added:

(18) $$L_{traj}=\frac{1}{\left|T\right|}\sum_{\left(i,t\right)}\left(|\right|x_{i}^{t}-T_{t\leftarrow t-1}x_{i}^{t-1}{\left|\right|}_{1}+\beta \cdot |r_{i}^{t}-r_{i}^{t-1}\left|\right)$$

where T is the set of matching points on the same trajectory in consecutive frames; |T| is the total number of trajectory points; *i* is the trajectory point index; *t* is the current-frame time; $x_{i}^{t}$ and $x_{i}^{t-1}$ are the 3D coordinate vectors of the *i*-th trajectory point in the current frame and the previous frame, respectively; $T_{t\leftarrow t-1}$ is the pose transformation matrix from the previous frame coordinate system to the current frame coordinate system; ||·|| is the L1 norm, used to calculate the sum of the absolute values of the coordinate differences; $r_{i}^{t}$ and $r_{i}^{t-1}$ represent the reflectance values of the *i*-th trajectory point in the current frame and the previous frame, respectively; and beta is the weight controlling the reflectance term. Finally, the total loss of the TCSP module is composed of two parts:

(19) $$L_{TCSP}=\lambda_{rank}L_{rank}+\lambda_{traj}L_{traj}$$

where $\lambda_{rank}$ and $\lambda_{rank}$ are loss weight hyperparameters.

**BCAF Module Loss.** The BCAF module achieves efficient interaction between camera BEV features and LiDAR BEV features through a bidirectional cross-modal attention mechanism. Let the attention matrix from the camera to the LiDAR branch be A and the attention matrix from the LiDAR to the camera branch be A. They are normalized to A. To ensure the symmetry of the cross-modal interaction, an attention consistency loss is introduced:

(20) $$L_{att}=\left|\right|{\tilde{A}}_{c\to l}-{\tilde{A}}_{l\to c}{\left|\right|}_{1}$$

where ${\tilde{A}}_{c\to l}$ is the normalized attention matrix from the camera to the LiDAR, ${\tilde{A}}_{l\to c}$ is the normalized attention matrix from the LiDAR to the camera, and ||·|| is the L1 norm. The fused BEV features are $F_{c2l}$ and $F_{l2c}$, respectively. To reduce feature shifts between modalities, a feature consistency loss is introduced:

(21) $$L_{feat}=\frac{1}{\left|\Omega \right|}\sum_{g\in \Omega }\left|\right|F_{c2l}\left(g\right)-F_{l2c}\left(g\right){\left|\right|}_{1}$$

where Ω is the set of BEV grid coordinates; |Ω| is the total number of BEV grids; g is a position index in the grid; $F_{c2l}\left(g\right)$ and $F_{l2c}\left(g\right)$ are the feature vectors from $F_{c2l}$ and $F_{l2c}$, respectively, at BEV grid position g; and ||·|| is the L1 norm. Finally, the total loss of the BCAF module is

(22) $$L_{BCAF}=\lambda_{att}L_{att}+\lambda_{feat}L_{feat}$$

where $\lambda_{att}$ and $\lambda_{feat}$ are loss weight hyperparameters.

**Detection Loss.** The loss function for this model in 3D detection tasks consists of two parts: object classification loss and 3D bounding box regression loss. The classification loss component uses standard cross-entropy loss to measure the difference between the predicted category probabilities and the true labels. Let the total number of training samples be *N*, the total number of categories be *C*, the predicted category probability of the *i*-th sample be ${\hat{p}}_{ic}$, and the one-hot encoding of the true category label be $y_{ic}$; then, the classification loss is defined as

(23) $$L_{cls}=-\frac{1}{N}\sum_{i=1}^{N}\sum_{c=1}^{C}y_{ic}log\left({\hat{p}}_{ic}\right)$$

This loss can effectively optimize the network’s category discrimination ability and maintain good stability even in scenarios with a large number of categories or uneven sample distribution.

The bounding box regression loss is used to optimize the consistency between the predicted box and the true box in terms of position and scale. In this paper, the L1 loss is chosen to measure the deviation between the two. Let $N_{pos}$ be the number of positive samples, and let $b_{i}$ and $b_{i}^{*}$ be the predicted bounding box parameters and true parameters of the *i*-th positive sample, respectively. Then, the L1 loss is defined as

(24) $$L_{box}=\frac{1}{N_{pos}}\sum_{i=1}^{N_{pos}}\left|\right|b_{i}-b_{i}^{*}{\left|\right|}_{1}$$

where the bounding box parameter $b_{i}$ includes the center coordinates, 3D dimensions, and orientation. The L1 loss can directly minimize the absolute error between the predicted value and the actual value, thereby improving the accuracy of target localization. Therefore, the total loss in the detection section is

(25) $$L_{dect}=\lambda_{cls}L_{cls}+\lambda_{box}L_{box}$$

where $\lambda_{cls}$ and $\lambda_{box}$ are the weight coefficients for classification and regression loss, respectively.

The final total loss of SETR-Fusion consists of the following four parts:

(26) $$L_{all}=L_{dect}+L_{DSSA}+L_{TCSP}+L_{BCAF}$$

## 4. Experiments

### 4.1. Datasets and Metrics

We perform experimental validation on nuScenes [24], an authoritative large-scale multimodal 3D target detection dataset in the field of autonomous driving, which contains 1000 finely labeled real driving scenarios, including 700 training, 150 validation, and 150 test sets, respectively, with an observation duration of about 20 s for each scenario. The data are captured by 6 cameras with a resolution of 1600×900, one 32-line LiDAR with a scanning frequency of 20 Hz, and five millimeter-wave radars in a 360-degree field of view. The dataset provides 3D bounding box truths by labeling keyframes at a frequency of 2 Hz, covering 10 categories, such as vehicles, pedestrians, etc., and the setup is equipped with sensor calibration tools to achieve cross-modal data alignment. We used the official evaluation metrics of nuScenes, which include the nuScenes detection score (NDS), mean accuracy (mAP), mean translation error (mATE), mean scale error (mASE), mean orientation error (mAOE), mean velocity error (mAVE), and mean attribute error (mAAE). The mAP is the average accuracy calculated from the threshold distance of 0.5 m/1 m/2 m/4 m from the center of the BEV. The NDS is a comprehensive index for the evaluation of the detection performance and is calculated as follows:

(27) $$NDS=\frac{1}{10}\left[5\times mAP+\sum_{mAP\in TP}\left(1-min\left(1,mTP\right)\right)\right]$$

where TP is the set of all true metrics, and TP={mATE,mASE,mAOE,mAVE,mAAE}. mTP denotes the average of all categories. Higher values of mAP and NDS indicate better model performance. Meanwhile, lower values of mATE, mASE, mAOE, mAVE, and mAAE indicate better performance.

In addition to the conventional metrics mentioned above, we also conducted a statistical significance analysis. Specifically, all experiments were repeated under multiple random seeds, with the results reported as the mean ± standard deviation. To further validate whether the observed improvements were statistically significant rather than random fluctuations, this study performed a two-sample *t*-test between the proposed method and the baseline method. The test statistic was defined as

(28) $$t=\frac{{\bar{x}}_{1}-{\bar{x}}_{2}}{\sqrt{\frac{s_{1}^{2}}{n_{1}}+\frac{s_{2}^{2}}{n_{2}}}}$$

where ${\bar{x}}_{1}$ and ${\bar{x}}_{2}$ are the means of the two groups, $s_{1}^{2}$ and $s_{2}^{2}$ are the unbiased variances, and $n_{1}$ and $n_{2}$ are the sample sizes. We report the corresponding *p*-values, with p<0.05 considered statistically significant.

### 4.2. Implementation Details

The implementation of SETR-Fusion is based on the open-source detection framework mmDetection3D (v1.1.0) [63] and Pytorch (v1.10.0) [64] with Python 3.8.20 and CUDA 11.3. The model is trained on 8 NVIDIA L40 GPUs, with a per-GPU batch size of 8, resulting in a global batch size of 64. For the camera branch trunk, we extract image features with Swin Transformer [57] and image semantic features with U-Net [56], and we perform multiscale feature fusion using the FPN [58], with the image size input resolution set to 256×704. For the LiDAR branch trunk, the experiments are performed by using VoxelNet [2] to voxelize the irregular point cloud, with the voxel size set to (0.075 m, 0.075 m, 0.2 m), and the detection distance is set as follows: X-axis and Y-axis [−54 m, 54 m] and Z-axis [−5 m, 3 m]. Then, 3D sparse convolution [3] is used to extract 3D point cloud features. The optimization process uses the AdamW [65] optimizer with the weight decay factor set to $10^{-1}$. We adopt the classic two-stage training paradigm: (1) we first pre-train the LiDAR branch and the vision branch independently and separately to complete the optimization of the modality-specific feature extractor; (2) we load frozen pre-training encoder weights and fine-tune the multimodal fusion module jointly from end to end to achieve the co-optimization of cross-modal feature interaction. The single-model prediction results are directly outputted in the inference stage without the need for test-time enhancement or additional post-processing, which ensures real-time performance while maintaining the reproducibility of the results. The size of the BEV feature map is set to 200×200, and the mesh resolution is 0.512 m.

### 4.3. Comparison of Results

**Figure 9:**
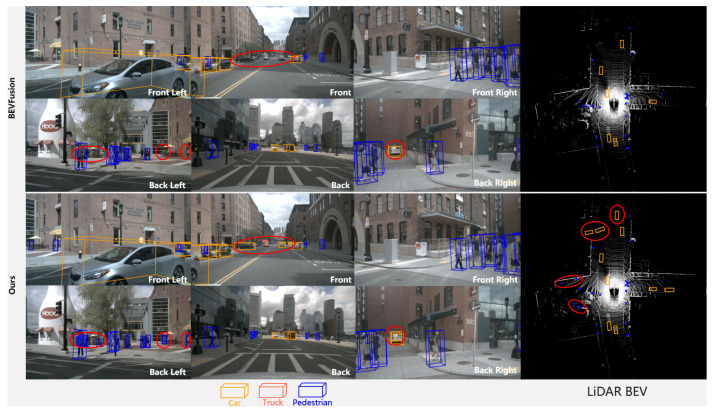
Visualization of target detection qualitative results. The top is the visualization of the baseline model, and the bottom is the visualization of our model. The red circles highlight the differences in the detection results between the two types of methods. We observe that SETR-Fusion detects distant objects as well as improving missed detections, a comparative result that proves the reliability of our approach.

**Figure 10:**
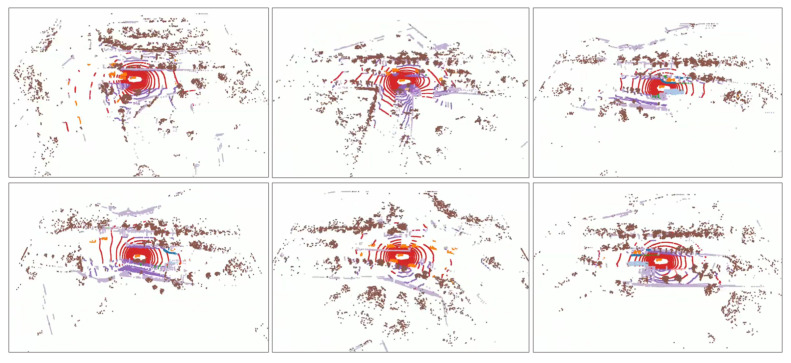
Point cloud visualization results after semantic processing. We endowed the point cloud with semantic information and also filtered the error points based on temporal consistency. Different colors in the figure correspond to different categories of objects.

The comparison of the results with those of previous unimodal target detection and multimodal target detection methods on the nuScenes dataset is demonstrated in Table 1, where our method achieves values of 71.2% mAP and 73.3% NDS on the test set of the nuScenes dataset, which is a significant improvement from the unimodal 3D target detection method. In addition, compared to the baseline method, the mAP and NDS values on our value set increase by 1.0% and 0.4%, respectively, and the detection values for small object categories such as Motor, Bike, Ped, and T.C. increase by 1.2%, 2.9%, 1.3%, and 0.5%, respectively. Based on the improvement in the detection performance, our SETR-Fusion has strong advantages in the small object detection context. The target detection qualitative results are demonstrated in Figure 9, and the semantically enhanced point cloud visualization results are demonstrated in Figure 10, where SETR-Fusion shows a significant improvement for the detection of distant and small instances, as well as miss detection. In Table 2, we provide specific metrics for the computational efficiency of our model. The results show that, compared to the baseline model, our model increases the number of parameters by approximately 11%, FLOPs by approximately 15%, the memory overhead by approximately 22%, and latency by approximately 23 ms/frame. Nevertheless, our method achieves a +1.0% improvement in mAP and a +0.4% improvement in the NDS, with notable gains in long-range and small-object scenarios. Our approach achieves a reasonable trade-off between accuracy and efficiency while maintaining deployability. We attribute this gain to the effective utilization of high-level semantic information to address the heterogeneity of point clouds and images in feature representation and to guide finer fusion, improving the point cloud’s shortcomings at the fine-grained level.

**Figure 11:**
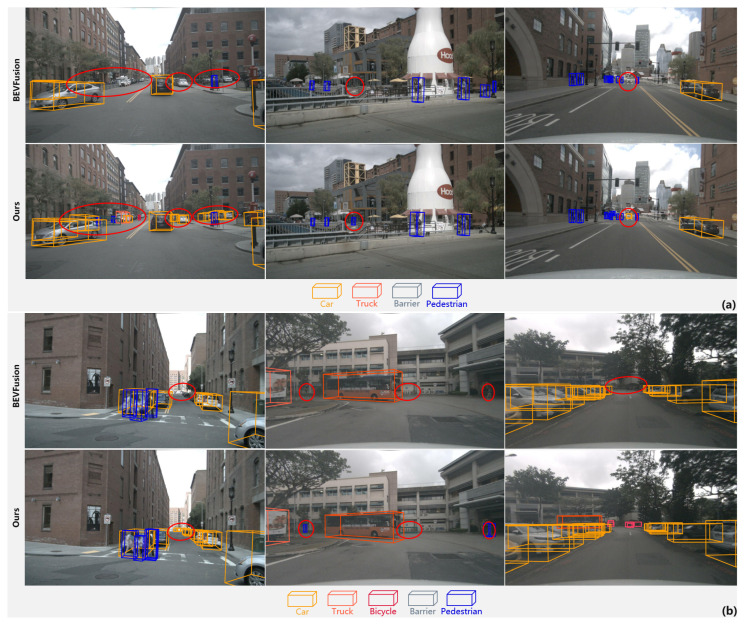
The 3D detection performance for distant and small objects. We selected several scenes containing distant and small objects and conducted a more extensive and detailed comparison between the model and the baseline detection performance. Subfigure (**a**) Detection performance comparison for Scenario 1; Subfigure (**b**) Detection performance comparison for Scenario 2. Qualitatively, we can observe that this model does indeed show a significant improvement in its ability to detect distant and small objects.

In order to better evaluate the model’s performance, we divided the detection range (0–54 m) into three regions—close range (0–18 m), medium range (18–36 m), and long range (36–54 m)—and compared SETR-Fusion with the baseline model in terms of the official nuScenes evaluation metrics. We specifically compared the small target objects detected at different distances in terms of the AP differences, as shown in Table 3 and Table 4, respectively. The results in Table 2 show that SETR-Fusion achieves optimal performance at all ranges regarding the mAP and NDS, with a particularly significant improvement at long distances: the mAP is 2.49% higher than that of BEVFusion, and the NDS is 2.25% higher. The results in Table 4 show that the SETR-Fusion model exhibits a significant advantage over BEVFusion on the small target detection task. This advantage is most prominent in the long-range metric, which represents the small-target scene. At this range, objects with a small pixel share and little detailed information are the most difficult to detect. The small-object AP metric of SETR-Fusion is at least 0.76% higher than that of the baseline at this range. To better demonstrate the detection advantage of the SETR-Fusion model for distant small objects, we selected some scenes with distant small objects for comparison, as shown in Figure 11, which clearly indicates its stronger overall detection capability for small objects. STER-Fusion’s strengths stem from the cross-modal semantic interaction mechanism, which fuses the precise geometric positioning of LiDAR with the rich semantic information of the camera to optimize target candidate screening, classification regression, and category priors through semantic guidance, significantly improving the localization and scale estimation accuracy. Multimodal complementarity further strengthens the performance: the close range relies on the dense LiDAR point cloud to achieve high-precision localization, while the long range utilizes the relatively dense ray geometry information of the camera to effectively compensate for the lack of direction estimation of the sparse LiDAR point cloud. This validates the robustness of the fusion strategy in cross-distance scenarios.

### 4.4. Ablation Studies

In this section, we present ablation experiments and analyze in depth the key components of SETR-Fusion, including the multimodal ablation, DSSA, TCSP, and BCAF modules. The mAP and NDS are computed on the nuScenes validation set.

**Multiple modalities.** Multimodal fusion exhibits the following improvements, as seen in Table 5: the mAP improves by 6.57% and the NDS improves by 6.99% with respect to the pure LiDAR method, and the mAP improves by 33.15% and the NDS improves by 30.57% with respect to the pure camera method.

**Component ablation.** To illustrate the effectiveness of our designed modules, we performed ablation experiments on the main modules, DSSA, TCSP, and BCAF, as shown in Table 6. It can be seen that our DSSA module improves the baseline mAP by 0.04% and the NDS by 0.11%; TCSP improves the baseline mAP by 0.46% and the NDS by 0.26%; and BCAF improves the baseline mAP by 0.42% and the NDS by 0.14%. In addition, the combination of the DSSA module and the TCSP module improves the baseline mAP by 1.25% and the NDS by 1.41%; the combination of the DSSA module and the BCAF module improves the baseline mAP by 1.58% and the NDS by 1.10%; and the combination of the TCSP module and the BCAF module improves the baseline mAP by 1.64% and the NDS by 1.31%. This simultaneous, synergistic improvement in performance when utilizing the three module designs leads to a combined improvement of 2.11% in the mAP and 1.79% in the NDS.

**Ablation study of feature fusion methods.** In the feature fusion module, an ablation study enables us to explore whether the fusion strategy is effective or not. As shown in Table 7, in this experiment, we set up five fusion mechanisms, i.e., Add, Concat, cross-attention from image to LiDAR, cross-attention from LiDAR to image, and the BCAF module. Comparing the one-sided cross-attention mechanisms, it can be found that the detection results obtained with LiDAR as a query are better than with the image as a query, because the point cloud can provide more accurate geometric information than the camera. The BCAF module, on the other hand, significantly improves the multimodal perception performance over the unimodal scheme by simultaneously using both the LiDAR and image as the query and fusing the complementary advantages of the two.

### 4.5. Module Effectiveness Analysis

To further evaluate whether the observed gains with DSSA, TCSP, and BCAF fall within the range of statistical noise, we reran the experiments using five different random seeds and report the average results with standard deviations. The results are shown in Table 8. Although DSSA exhibits a smaller average gain, its improvements remain consistent and statistically significant (*p* < 0.05) across different seeds. TCSP and BCAF deliver more substantial performance enhancements, both achieving highly significant *p*-values (*p* < 0.001). This confirms that the improvements are not random fluctuations but robust effects generated by our proposed modules.

Among them, TCSP contributed the greatest gains, achieving average improvements of +0.46 in the mAP and +0.26 in the NDS, indicating that temporal consistency is crucial for stable cross-frame point–semantic associations. BCAF further enhanced cross-modal interactions, yielding average gains of +0.42 in the mAP and +0.14 in the NDS. Collectively, these results validate the effectiveness of all three modules and highlight their complementary contributions.

### 4.6. Efficiency–Accuracy Trade-Offs of Fusion Choices

To evaluate the trade-off between efficiency and accuracy across different multimodal fusion strategies, we conducted experiments based on the anchor configuration—specifically, models with DSSA and TCSP enabled. We compared the default concatenation fusion scheme with alternative designs: element-wise addition, two types of unidirectional dense cross-attention, bidirectional dense cross-attention, and our proposed bidirectional low-rank cross-attention. Table 9 reports the incremental cost relative to the anchors and comprehensive metrics on the nuScenes validation set. The results indicate that Add fusion is marginally more efficient than Concat fusion; both unidirectional cross-attention types significantly increase the floating-point operations and introduce 14 ms latency, while bidirectional dense cross-attention doubles the computational burden and adds 32 ms. In contrast, our low-rank design reduces the parameters and FLOPs by over 40% compared to dense bidirectional attention, lowering the per-frame latency from 143 ms to 127 ms while maintaining the accuracy advantages described in Section 4.3. These results validate that our BCAF module achieves a reasonable trade-off between efficiency and accuracy, demonstrating the validity of employing low-rank projections in both attention directions.

### 4.7. Sensitivity Analysis of Loss Weights

To validate the robustness of the loss function design, we conducted sensitivity analyses on the weighting factors of each loss term, including the foreground–background saliency loss weight, temporal–semantic fusion loss weight, and bidirectional cross-attention loss weight. While keeping other weights constant, we adjusted each coefficient within the ranges of 0.5×, 1×, and 2× relative to the default setting.

As shown in Table 10, the detection performance remained stable under these moderate weight variations. Specifically, the average AP values and NDS performance across all test scales fluctuated within ±0.3%. This indicates that the model exhibits low sensitivity to precise weight adjustments, suggesting that the performance gains primarily stem from the innovative module design rather than hyperparameter fine-tuning.

## 5. Discussion

Despite SETR-Fusion’s outstanding performance in perception, the current model still has certain limitations. The first limitation lies in the computational efficiency and real-time performance. The current inference speed is slower than those of some efficiency-oriented baselines because the deep interaction and feature enhancement modules add computational complexity. The model’s real-time performance lags behind that of certain baselines, and its computational complexity poses challenges for high-frame-rate scenarios and in-vehicle deployment, necessitating efficiency optimization while maintaining accuracy. Secondly, the model relies on image semantic information to enhance the detection capabilities for small objects, which may compromise the detection performance in dark night environments, under strong light interference, or in extreme occlusion scenarios. Additionally, our model depends on precise camera–LiDAR calibration. As a multimodal detection framework, its feature alignment in the BEV space heavily relies on precise extrinsic parameters. When the calibration parameters drift, the resulting cross-modal misalignment can propagate through the processing pipeline: BCAF experiences degraded geometric–semantic correspondence, DSSA suffers from the reduced reliability of projection supervision signals due to boundary mismatches, and TCSP’s temporal consistency is compromised by accumulated alignment errors.

In our future work, we will focus on model lightweighting and acceleration, designing more efficient interaction mechanisms, incorporating hardware-cooperative optimization, and exploring temporal modeling and multitask applications. We are committed to enhancing the model’s robustness in long-tail scenarios such as extreme weather, lighting conditions, and severe occlusion. Through targeted data augmentation, domain adaptation techniques, or uncertainty modeling, we aim to ensure model reliability and safety in complex real-world open-road environments. Simultaneously, we plan to explore calibration error-resistant fusion strategies to reduce the dependence on out-of-calibration parameters. Moreover, our bidirectional cross-attention and temporal consistency modules demonstrate significant potential beyond autonomous driving, extending to intelligent transportation systems (ITSs). For instance, in drone aerial surveillance tasks, the temporal consistency mechanism reduces false alarm rates and enhances the tracking accuracy for small targets in dense traffic environments—aligning closely with recent ITS research [68]. Therefore, extending this framework to drone video stream analysis represents a highly valuable future research direction.

## 6. Conclusions

Aiming at the problem of the insufficient utilization of semantic information and an insufficient interaction depth in the fusion of LiDAR and camera features in the current mainstream BEVFusion method, this paper comprehensively analyzes the complementary characteristics of LiDAR features and camera features. Based on this, we propose the SETR-Fusion method. The core of the method lies in double optimization: on the one hand, we strengthen semantic extraction in LiDAR point clouds and enhance the geometric perception of camera features, thereby compensating for the limitations of each modality and improving semantic representation; on the other hand, in the key BEV spatial feature fusion stage, the shallow or unidirectional interaction mode is abandoned, and the bilateral interaction mechanism is innovatively introduced, which realizes the LiDAR’s accurate and precise interaction with the camera. By effectively combining and amplifying the complementary advantages of the two modalities under the unified BEV framework, SETR-Fusion significantly outperforms the existing optimal methods on the authoritative nuScenes 3D target detection benchmark, which fully validates the effectiveness of the dual optimization strategy.

## Figures and Tables

**Table 1:** Comparison with other methods on the nuScenes test set. “L” is LiDAR and “C” is camera. C.V., Ped, and T.C. denote construction vehicles, pedestrians, and traffic cones, respectively. The best results are shown in bold.

Method	Modality	mAP	NDS	Car	Truck	C.V.	Bus	Trailer	Barrier	Motor	Bike	Ped	T.C.
BEVDet [28]	C	42.4	48.8	64.3	35.0	16.2	35.8	35.4	61.4	44.8	29.6	41.1	60.1
DETR3D [9]	C	41.2	47.9	60.3	33.3	17.0	29.0	35.8	56.5	41.3	30.8	45.5	62.7
BEVFormer [29]	C	48.1	56.9	67.7	39.2	22.9	35.7	39.6	62.5	47.9	40.7	54.4	70.3
PointPillars [4]	L	30.5	45.3	68.4	23.0	4.1	28.2	23.4	38.9	27.4	1.1	59.7	30.8
CenterPoint [37]	L	60.3	67.3	85.2	53.5	20.0	63.6	56.0	71.1	59.5	30.7	84.6	78.4
PointPainting [14]	C + L	46.4	58.1	77.9	35.8	15.8	35.2	37.3	60.2	41.5	24.1	73.3	62.4
MVP [48]	C + L	66.4	70.5	86.8	58.5	26.1	67.4	57.3	74.8	70.0	49.3	89.1	85.0
AutoAlign [66]	C + L	65.8	70.9	85.9	55.3	29.6	67.7	55.6	-	71.5	51.5	86.4	-
AutoAlignV2 [67]	C + L	68.4	72.4	87.0	59.0	33.1	69.3	59.3	-	72.9	52.1	87.6	-
TransFusion [13]	C + L	68.9	71.7	87.1	60.0	33.1	68.3	60.8	78.1	73.6	52.9	88.4	86.7
BEVFusion [16]	C + L	69.2	71.8	88.1	60.9	34.4	69.3	62.1	78.2	72.2	52.2	89.2	85.2
BEVFusion [17]	C + L	70.2	72.9	88.6	60.1	39.3	69.1	63.8	**80.0**	74.1	51.0	89.2	86.5
CSDSFusion [48]	C + L	70.5	72.6	89.1	62.6	39.0	**71.8**	63.0	79.9	73.3	51.9	88.3	85.8
GraphBEV [43]	C + L	70.9	73.2	89.9	**63.1**	**40.0**	71.4	**64.2**	79.5	74.2	52.8	89.1	86.4
SETR-Fusion (ours)	C+L	**71.2**	**73.3**	**90.8**	62.3	38.5	70.6	63.5	79.6	**75.3**	**53.9**	**90.5**	**87.0**

**Table 2:** Comparison of parameters, FLOPs, memory, latency, and FPS on the nuScenes validation set. All metrics were obtained from inference statistics on a single RTX 3090 GPU.

Model	Params (M)	FLOPs (G/frame)	Memory (GB)	Latency (ms/frame)	FPS	mAP (%)	NDS (%)
BEVFusion (baseline)	40.84	253.2	9.1	104.17	9.60	70.2	72.9
SETR-Fusion (ours)	45.30 (+11%)	291.0 (+15%)	11.1 (+22%)	127.00	7.87	71.2	73.3

**Table 3:** Comparison of metrics for different distances on the nuScenes validation set. Metrics are taken from the official nuScenes evaluation metrics, and the range is defined as the distance from the self-vehicle to the center of the object, with the best results shown in bold.

Range	Method	mAP	NDS	mATE	mASE	mAOE	mAVE	mAAE
Near	BEVFusion	76.23	76.07	23.12	24.33	24.02	22.35	**26.65**
	SETR-Fusion	**78.10**	**77.37**	**21.93**	**22.75**	**22.84**	**19.82**	29.48
Middle	BEVFusion	65.87	69.10	29.49	23.19	**31.72**	26.41	**27.54**
	SETR-Fusion	**67.01**	**69.14**	**28.68**	**23.30**	34.61	**25.39**	31.66
Far	BEVFusion	35.65	48.56	51.29	45.00	49.31	**30.60**	**16.42**
	SETR-Fusion	**38.14**	**50.04**	**49.47**	**43.51**	**47.01**	32.14	18.16
Whole	BEVFusion	67.17	70.57	28.55	25.63	30.76	25.12	**20.07**
	SETR-Fusion	**69.28**	**72.25**	**26.21**	**24.21**	**29.43**	**23.59**	20.50

**Table 4:** Comparison of AP metrics on the nuScenes validation set for small targets at different distances. Metrics are taken from the official evaluation metrics of nuScenes. The range is defined as the distance from the self-vehicle to the center of the object, and the best results are shown in bold.

Range	Method	mAP	Motor	Bike	Ped.	T.C.	Car
Near	BEVFusion	76.23	76.07	56.81	**91.94**	87.35	90.76
	SETR-Fusion	**78.10**	**77.37**	**60.02**	90.56	**89.17**	**91.29**
Middle	BEVFusion	65.87	69.10	48.77	79.36	77.94	81.75
	SETR-Fusion	**67.01**	**69.14**	**50.38**	**80.40**	**79.53**	**82.47**
Far	BEVFusion	35.65	48.56	27.53	44.76	51.26	64.55
	SETR-Fusion	**38.14**	**50.04**	**28.61**	**47.93**	**52.27**	**65.31**
Whole	BEVFusion	67.17	70.57	45.35	85.01	80.39	83.58
	SETR-Fusion	**69.28**	**72.25**	**48.92**	**87.50**	**81.44**	**85.52**

**Table 5:** Multimodal ablation experiments. “↑” indicates that the higher the value, the better the model’s detection performance. “” indicates that the model has adopted this mode.

LiDAR	Camera	mAP↑	NDS↑
✓		62.71	65.26
	✓	36.13	41.68
✓	✓	69.28	72.25

**Table 6:** Component ablation experiments. “↑” indicates that the higher the value, the better the model’s detection performance. “✓” indicates that the model has adopted this module.

ID	DSSA	TCSP	BCAF	mAP↑	NDS↑
(1)				67.17	70.38
(2)	✓			67.21	70.49
(3)		✓		67.63	70.64
(4)			✓	67.59	70.52
(5)	✓	✓		68.42	71.19
(6)	✓		✓	68.75	71.48
(7)		✓	✓	68.81	71.69
(8)	✓	✓	✓	69.28	72.25

**Table 7:** Evaluating the impact of BCAF. “↑” indicates that the higher the value, the better the model’s detection performance.

Fusion Method	mAP↑	NDS↑
Add	66.43	69.66
Concat	68.42	71.19
Cross/Image	68.32	71.26
Cross/LiDAR	68.74	71.81
BCAF	69.28	72.25

**Table 8:** Statistical significance of DSSA, TCSP, and BCAF modules. Mean ± std performance improvements across 5 seeds and paired *t*-test results on nuScenes validation set. “↑” indicates that the higher the value, the better the model’s detection performance.

Module	mAP↑ (Mean ± std)	NDS↑ (Mean ± std)	*p*-Value (mAP)	*p*-Value (NDS)
DSSA	+0.04±0.02	+0.11±0.03	0.011	0.0012
TCSP	+0.46±0.04	+0.26±0.05	$1.4\times 10^{-5}$	$3.1\times 10^{-4}$
BCAF	+0.42±0.03	+0.14±0.04	$6.2\times 10^{-6}$	0.0014

**Table 9:** Comparison of operational efficiency across integration methods. The * denotes dense cross-attention without low-rank projection.

Method	r	Params (M)	FLOPs (G/frame)	Memory (GB)	Latency (ms/frame)	FPS
Add	-	43.21 (−0.13)	260.3 (−4.25)	9.7 (−0.10)	110.5 (−0.5)	9.05
Concat	-	43.34	264.6	9.8	111.0	9.01
Cross-Attn * (Cam→LiDAR)	-	49.84 (+6.50)	286.0 (+21.4)	10.7 (+0.88)	124.6 (+13.6)	8.02
Cross-Attn * (LiDAR→Cam)	-	49.84 (+6.50)	287.2 (+22.6)	10.7 (+0.91)	125.4 (+14.4)	7.98
Bidirectional Cross-Attn *	-	56.44 (+13.10)	308.6 (+44.0)	11.6 (+1.80)	143.0 (+32.0)	6.99
Bidirectional Cross-Attn	64	50.74 (+7.40)	291.6 (+27.0)	10.9 (+1.10)	127.0 (+16.0)	7.87

**Table 10:** Comparison of weight factor sensitivity.

Module	λ	mAP (%)	NDS (%)
DSSA	0.5×	69.16	72.08
	1×	69.28	72.25
	2×	69.17	72.14
TCSP	0.5×	69.05	72.03
	1×	69.28	72.25
	2×	69.11	72.12
BCAF	0.5×	69.10	72.06
	1×	69.28	72.25
	2×	69.09	72.10

## Data Availability

The data presented in this study are available in nuScences dataset at https://www.nuscenes.org/ (accessed on 9 August 2025), and reference [24].

## Abbreviations

The following abbreviations are used in this manuscript:

3D	Three-Dimensional

LiDAR	Light Detection and Ranging
BEV	Bird’s Eye View
TCSP	Temporally Consistent Semantic Point Fusion
DSSA	Discriminative Semantic Saliency Activation
BCAF	Bilateral Cross-Attention Fusion
SETR-Fusion	Semantic-Enhanced and Temporally Refined Bidirectional BEV Fusion
FPN	Feature Pyramid Network
MLP	Multilayer Perceptron
FFN	Feedforward Network
BN	Batch Normalization
LN	Layer Normalization
GELU	Gaussian Error Linear Unit
LSS	Lift–Splat–Shoot
FRM	Foreground Response Map
DWM	Depth Weight Map
DA-FRM	Depth-Augmented Foreground Response Map
DA-BRM	Depth-Augmented Background Response Map
ReLU	Rectified Linear Unit
KD-Tree	K-Dimensional Tree
NDS	NuScenes Detection Score
mAP	Mean Accuracy
mATE	Mean Translation Error
mASE	Mean Scale Error
mAOE	Mean Orientation Error
mAVE	Mean Velocity Error
mAAE	Mean Attribute Error
AdamW	Adaptive Moment Estimation with Weight Decay
CUDA	Compute Unified Device Architecture
ITS	Intelligent Transportation System
